# Oral Rehabilitation and Management for Secondary Sjögren's Syndrome in a Child

**DOI:** 10.1155/2016/3438051

**Published:** 2016-11-27

**Authors:** Tatiana Kelly da Silva Fidalgo, Carla Nogueira, Marcia Rejane Thomas Canabarro Andrade, Andrea Graciene Lopez Ramos Valente, Patricia Nivoloni Tannure

**Affiliations:** ^1^Universidade do Estado do Rio de Janeiro, Boulevard Vinte e Oito de Setembro, 175 Vila Isabel, 21941-913 Rio de Janeiro RJ, Brazil; ^2^Universidade Salgado de Oliveira, Pólo Niterói, Rua Marechal Deodoro, 263 Centro, 24030-060 Niterói, RJ, Brazil; ^3^Universidade Veiga de Almeida, Rua Ibituruna 108, Tijuca, 20271-020 Rio de Janeiro, RJ, Brazil; ^4^Department of Specific Formation, School of Dentistry, Universidade Federal Fluminense, Rua Dr. Silvio Henrique Braune 22, 28625-650 Nova Friburgo, RJ, Brazil

## Abstract

The aim of this paper is to describe a rare case report of a pediatric patient with secondary Sjögren's syndrome (SSS). A 12-year-old female child was referred to the Pediatric Dentistry Clinic with the chief complaint of tooth pain, dry mouth, and tooth sensibility. The patient was submitted to orthodontic treatment prior to syndrome diagnosis. The clinical treatment consisted of the interruption of orthodontic treatment and restoring the oral condition with dental treatment and the use of artificial saliva in an innovative apparatus. Dental therapy involved the control of dental caries, periodontal disease, and opportunistic fungal infections and the use of fluoride-rich solutions. The present clinical case describes clinical and laboratory aspects of SSS in pediatric patients. The management of the oral findings promoted an improvement in the oral health status and quality of life of the child.

## 1. Introduction

Sjögren's syndrome (SS) is a chronic autoimmune disease of the exocrine glands characterized by focal lymphocytic infiltration and destruction of these glands [1]. SS may occur alone, as primary SS, or may accompany other autoimmune disorders as secondary Sjögren's syndrome (SSS) [2, 3]. SS typically occurs when xerostomia and xerophthalmia are present. The prevalence in the general population is about 0.5% to 3% [4]. Females are more affected, especially in the fourth and fifth decades of life. Children and young adults are rarely affected. Although it is not an inherited disease, there is evidence of a genetic influence. Keratoconjunctivitis sicca and xerostomia characterize the main clinical symptoms. Neurologic complications are probably underestimated and have been reported in 8% to 70% of Sjögren syndrome patients [1, 5, 6].

Primary disease is rare in childhood [7]. Parotid swelling was the most common symptom reported, such as dry eyes and xerostomia. Serological analysis showed positivity to rheumatoid factor in most of the cases and elevated positivity to antinuclear antibodies was also observed [8–10]. Oral alterations of SS include salivary gland dysfunction (diminished salivary flow), dental caries, stomatitis, and candidiasis, each of which negatively impacts the quality of life [10]. Saliva plays an important role in maintaining homeostasis in the oral cavity, preventing diseases in the hard and soft tissues [11–13]. Hyposalivation is generally accompanied by rapid progression of caries and the presence of candidiasis, consisting of major worsening of dental health [14, 15].

Therefore, this report describes a rare case of SSS that affected a 12-year-old girl submitted to an orthodontic treatment prior to the diagnosis. The case describes severe tooth destruction with oral rehabilitation and hyposalivation management.

## 2. Case Report

A 12-year-old female patient was referred to the Pediatric Dentistry Clinic, complaining of dry mouth, tooth sensibility, and dental pain. The patient reported three episodes of parotid gland enlargement. Her medical history included rheumatoid arthritis diagnosed 6 months before consultation. Regarding family history, her mother reported that both she and her sister presented symptoms of autoimmune diseases. In the current case report, the diagnosis of Sjögren's syndrome was confirmed by anti-Ro/anti-La antibodies, magnetic resonance imaging of the parotid and sublingual salivary glands, and also parotid contrast sialography. The patient was monitored by a doctor in regular appointments for the control of Sjögren's syndrome and rheumatoid arthritis and the treatment consisted of corticoid therapy.

**Figure 1:**
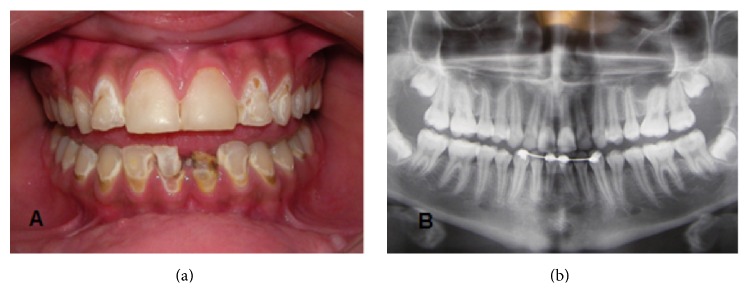
(a) The initial clinical appearance demonstrating carious lesions on buccal regions; (b) radiographic examination showing the fixed lingual orthodontic appliance for treatment contention and carious lesions.

An extraoral investigation showed dry lips and no glandular enlargement. During the intraoral exam (Figure 1(a)), extensive caries, gingival inflammation, accumulation of biofilm, poor hygiene, and deficient tooth brushing were observed. The presence of a fixed lingual orthodontic appliance between elements 33 and 43 (Figure 1(b)) was noted. The intraoral examination also confirmed the dryness of the mucous membranes, reduced salivary flow, dry lips, and touch sensitivity response to clinical instruments. Salivary flow, which showed a severely reduced rate at rest (0.05 mL/min), confirmed the findings of the parotid sialography test.

The patient was instructed to use alcohol-free mouthwash and to replace her toothpaste and toothbrush with others with the following characteristics: fluoride toothpaste with low abrasion and a toothbrush with a small head and straight, soft bristles. In order to moisturize the lips, the patient was advised to use cocoa butter lipstick.

It was possible to observe caries lesions in both clinical (Figure 1(a)) and radiographic (Figure 1(b)) images. Elements 31 and 41 received endodontic treatment and restorations with composite resin until the definitive prosthetic rehabilitation. Teeth 13, 12, 11, 21, and 23 presented deficient proximal restorations and were restored. Premolars and molars 17, 25, 27, and 35 were restored due to caries lesions. Pulp capping was performed on element 37 with glass ionomer cement. The vestibular regions of 12, 13 22, 23, 35, 34, 32, 42, 43, and 44 presented structural loss and were restored. Teeth 46 and 47 received resin-based fissure sealants. Duraphat® fluoride varnish was applied in white spot vestibular lesions. This was effective in remineralizing teeth and controlling hypersensitivity.

**Figure 2:**
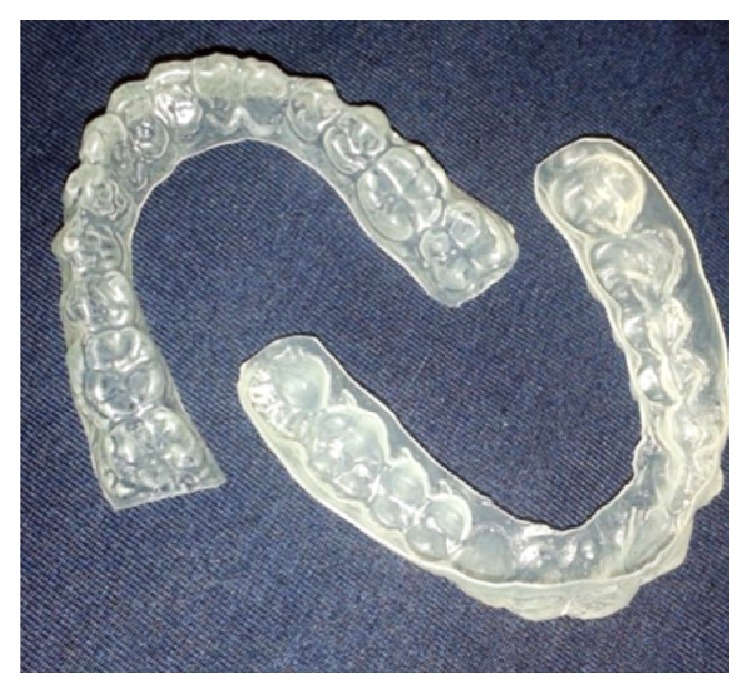
Tray for artificial saliva.

**Figure 3:**
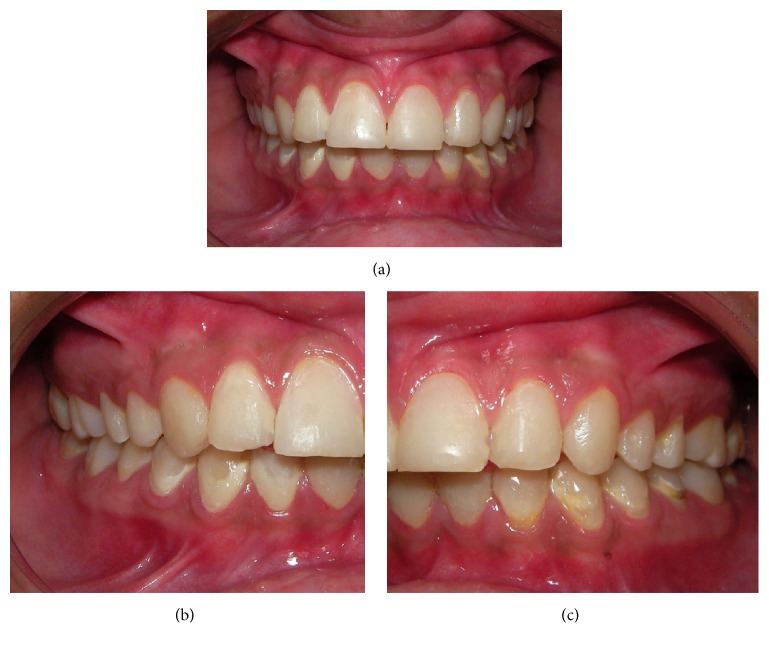
The clinical aspect after the restorative treatment (a). (b) and (c) are right and left sides, respectively.

To alleviate the xerostomia and manage the hyposalivation, artificial saliva was manipulated. We prepared a tray identical to those used in tooth whitening to be filled with artificial saliva (Figure 2) overnight. This procedure provided better hydration of the tissues of the oral cavity, in particular the oral mucosa, as shown in Figure 3.

## 3. Discussion

The preexistence of rheumatoid arthritis led to the classification of the patient as a case of SSS. Furthermore, the diagnosis was confirmed based on the most recent guidelines [16, 17]. In addition, the revised rules proposed by the Euro-American Group Consensus Criteria for the Classification of Sjögren's Syndrome were followed [17], which introduced more clearly defined rules to classify patients between primary and secondary types.

In the current case report, the patient reported autoimmune diseases in her family history. SSS presents in association with other autoimmune diseases [17]. In primary Sjögren's syndrome, clinical manifestations are limited to exocrine gland dysfunction while the secondary subtype of the disease involves the presence of other autoimmune diseases [18, 19]. Recurrent parotid gland enlargement often shows up as the first symptom of the disease in pediatric cases, followed by xerophthalmia and xerostomia. The occurrence of SSS in children and adolescents is not common and is often undiagnosed due to the limited applicability of the diagnostic criteria in pediatric patients [9, 17–19].

Decreased salivary secretion can lead to major changes in the oral mucosa, difficulty in swallowing and speaking, a sensation of burning in the mouth, and an increase in dental caries, but also to greater susceptibility to developing periodontal diseases [15]. Periodontal alterations were not present in the patient in the case reported here, perhaps due to her young age. The patient's condition was aggravated by poor oral hygiene that, combined with the use of an orthodontic appliance prior to the diagnosis of SSS, facilitated the occurrence of multiple carious lesions, which led to the need for endodontic procedures in several teeth.

The treatment of xerostomia in these patients is basically supportive therapy, with the aim of stimulating saliva production. In treating the oral symptoms of patients with respect to xerostomia, the use of artificial saliva and chewing gum without sugar is often indicated [20]. Also, the use of pilocarpine, a parasympathomimetic drug with effects similar to acetylcholine, is able to increase the production of secretions from exocrine glands in the body [21, 22]. Pilocarpine is usually used in the treatment of patients with hyposalivation that may occur as a side effect of radiation therapy for the head and neck for the treatment of cancer, but its use should also be indicated in some other special cases [21]. In the current case report, the patient was monitored by a doctor who controlled the disease using corticoid therapy and did not recommend pilocarpine, but an increase of water consumption. Low-level laser therapy has been proven to be an alternative to reducing the xerostomia, pain, and facial edema [23]. Artificial saliva is often used due to its minimal restrictions. Despite the widespread use of artificial saliva to treat hyposalivation and xerostomia [24], there are no alternatives to resolving this diminished flow rate during the night, when it is drastically reduced. Thus, we opted to produce a tray similar to a tooth-whitening tray for the application of artificial saliva for the maintenance of oral hydration, since the clearance of artificial saliva conventionally used as mouth rinse is fast and the tray was able to keep the artificial saliva surrounding the tissues. This alternative method allowed hydrating the hard and soft tissues not only during the day, but also for much of the night for a long time. This method has been proven to be satisfactory and is recommended, since it promotes prolonged contact of the artificial saliva with oral tissues, providing greater patient comfort and aiding in the control of new lesions. The patient was also instructed to brush her teeth with nonabrasive toothpaste and fluoride varnish was applied to white spot vestibular lesions in order to remineralize them and control the hypersensitivity. The inferior first molars received resin-based fissure sealants to prevent dental caries. Additionally, the patient presented a fixed lingual orthodontic appliance that increased the biofilm accumulation and the risk of caries. The contention was removed and the anterior teeth were submitted to restorative treatment. A knowledge of the systemic condition is essential to avoid treatments that bring about more losses than benefits, such as the use of orthodontic appliances. Therefore, dentists must indicate individually the treatments of each patient.

## 4. Conclusion

The dental therapeutic approach involved the control of caries, periodontal disease, and opportunistic fungal infections, oral hygiene instruction, the use of fluoride-rich solutions, the use of artificial saliva in a tray, and regular follow-up at short intervals. This approach proved to be effective in recovering the oral health and self-confidence and consequently improved the quality of life of the patient.

## References

[1] Qin B., Wang J., Yang Z. (2015). Epidemiology of primary Sjögren's syndrome: a systematic review and meta-analysis. *Annals of the Rheumatic Diseases*.

[2] Salliot C., Gottenberg J.-E., Bengoufa D., Desmoulins F., Miceli-Richard C., Mariette X. (2007). Anticentromere antibodies identify patients with Sjögren's syndrome and autoimmune overlap syndrome. *Journal of Rheumatology*.

[3] Meyer O. (2003). Evaluating inflammatory joint disease: how and when can autoantibodies help?. *Joint Bone Spine*.

[4] Fox R. I., Michelson P. (2000). Approaches to the treatment of Sjogren's syndrome. *The Journal of Rheumatology. Supplement*.

[5] Ramos-Casals M., Solans R., Rosas J. (2008). Primary Sjögren syndrome in Spain: clinical and immunologic expression in 1010 patients. *Medicine*.

[6] Michel L., Toulgoat F., Desal H. (2011). Atypical neurologic complications in patients with primary Sjögren's syndrome: report of 4 cases. *Seminars in Arthritis & Rheumatism*.

[7] Civilibal M., Canpolat N., Yurt A. (2007). A child with primary Sjögren syndrome and a review of the literature. *Clinical Pediatrics*.

[8] Shiboski S. C., Shiboski C. H., Criswell L. A. (2012). American College of rheumatology classification criteria for Sjögren's syndrome: a data-driven, expert consensus approach in the Sjögren's International Collaborative Clinical Alliance cohort. *Arthritis Care and Research*.

[9] Bowman S. J. (2002). Collaborative research into outcome measures in Sjogren's syndrome. Update on disease assessment. *Acta Rheumatologica Scandinavica. Supplementum*.

[10] de Souza T. R., Silva I. H. M., Carvalho A. T. (2012). Juvenile Sjögren syndrome: distinctive age, unique findings. *Pediatric Dentistry*.

[11] Fidalgo T. K. S., Freitas-Fernandes L. B., Almeida F. C. L., Valente A. P., Souza I. P. R. (2015). Longitudinal evaluation of salivary profile from children with dental caries before and after treatment. *Metabolomics*.

[12] Fidalgo T. K. S., Freitas-Fernandes L. B., Angeli R. (2013). Salivary metabolite signatures of children with and without dental caries lesions. *Metabolomics*.

[13] Fidalgo T. K. D. S., Freitas-Fernandes L. B., Ammari M., Mattos C. T., De Souza I. P. R., Maia L. C. (2014). The relationship between unspecific s-IgA and dental caries: a systematic review and meta-analysis. *Journal of Dentistry*.

[14] Pedersen A. M. L., Bardow A., Nauntofte B. (2005). Salivary changes and dental caries as potential oral markers of autoimmune salivary gland dysfunction in primary Sjögren's syndrome. *BMC Clinical Pathology*.

[15] Ravald N., List T. (1998). Caries and periodontal conditions in patients with primary Sjögren's syndrome. *Swedish Dental Journal*.

[16] Locht H., Pelck R., Manthorpe R. (2005). Clinical manifestations correlated to the prevalence of autoantibodies in a large (*n* = 321) cohort of patients with primary Sjögren's syndrome: a comparison of patients initially diagnosed according to the Copenhagen classification criteria with the American-European consensus criteria. *Autoimmunity Reviews*.

[17] Vitali C., Bombardieri S., Jonsson R. (2002). Classification criteria for Sjögren's syndrome: a revised version of the European criteria proposed by the American-European Consensus Group. *Annals of the Rheumatic Diseases*.

[18] Daniels T. E. (1996). Sjögren's syndrome: clinical spectrum and current diagnostic controversies. *Advances in Dental Research*.

[19] Nikitakis N. G., Rivera H., Lariccia C., Papadimitriou J. C., Sauk J. J. (2003). Primary Sjögren syndrome in childhood: report of a case and review of the literature. *Oral Surgery, Oral Medicine, Oral Pathology, Oral Radiology, and Endodontics*.

[20] Pinto A. (2014). Management of xerostomia and other complications of Sjögren's syndrome. *Oral and Maxillofacial Surgery Clinics of North America*.

[21] Ramos-Casals M., Tzioufas A. G., Stone J. H., Sisó A., Bosch X. (2010). Treatment of primary Sjögren syndrome: a systematic review. *Journal of the American Medical Association*.

[22] Brito-Zerón P., Sisó-Almirall A., Bové A., Kostov B. A., Ramos-Casals M. (2013). Primary Sjögren syndrome: an update on current pharmacotherapy options and future directions. *Expert Opinion on Pharmacotherapy*.

[23] Simões A., Platero M. D., Campos L., Aranha A. C., De Paula Eduardo C., Nicolau J. (2009). Laser as a therapy for dry mouth symptoms in a patient with Sjögren's syndrome: a case report. *Special Care in Dentistry*.

[24] Dost F., Farah C. S. (2013). Stimulating the discussion on saliva substitutes: a clinical perspective. *Australian Dental Journal*.

